# Aerosolized Antibiotics to Manage Ventilator-Associated Infections: A Comprehensive Review

**DOI:** 10.3390/antibiotics12050801

**Published:** 2023-04-23

**Authors:** Pavlos Myrianthefs, George E. Zakynthinos, Vasiliki Tsolaki, Demosthenes Makris

**Affiliations:** 1 “Agioi Anargyroi” General Hospital, School of Health Sciences, Department of Nursing, National and Kapodistrian University of Athens, 14564 Athens, Greece; 2 Third Cardiology Clinic, University of Athens, Sotiria Hospital, 11526 Athens, Greece; 3 Department of Intensive Care Medicine, University Hospital of Larissa, 41110 Larissa, Greece; 4 Faculty of Medicine, University of Thessaly, 41110 Larissa, Greece

**Keywords:** aerosolized antibiotics, intensive care unit, lower respiratory tract infections, nebulized antibiotics, tracheal tube colonization, treatment, ventilator-associated pneumonia, ventilator-associated tracheobronchitis

## Abstract

Background: Ventilator-associated lower respiratory tract infectious complications in critically ill patients cover a wide spectrum of one disease process (respiratory infection), initiating from tracheal tube and/or tracheobronchial colonization, to ventilator associated tracheobronchitis (VAT) and ventilator-associated pneumonia (VAP). VAP occurence has been associated with increased intensive care unit (ICU) morbidity (ventilator days, as well as length of ICU and hospital stay) and ICU mortality. Therefore, treatments that aim at VAP/VAT incidence reduction are a high priority. Aim: The aim of the present review is to discuss the current literature concerning two major aspects: (a) can aerosolized antibiotics (AA) administered in a pre-emptive way prevent the occurrence of ventilator-associated infections? and (b) can VAT treatment with aerosolized avert the potential evolution to VAP? Results: There were identified eight studies that provided data on the use of aerosolized antibiotics for the prevention of VAT/VAP. Most of them report favorable data on reducing the colonisation rate and the progression to VAP/VAT. Another four studies dealt with the treatment of VAT/VAP. The results support the decrease in the incidence to VAP transition and/or the improvement in signs and symptoms of VAP. Moreover, there are concise reports on higher cure rates and microbiological eradication in patients treated with aerosolized antibiotics. Yet, differences in the delivery modality adopted and resistance emergence issues preclude the generalisability of the results. Conclusion: Aerosolized antibiotic therapy can be used to manage ventilator-associated infections, especially those with difficult to treat resistance. The limited clinical data raise the need for large randomized controlled trials to confirm the benefits of AA and to evaluate the impact on antibiotic selection pressure.

## 1. Introduction

Lower respiratory tract infections are well known entities, including ventilator-associated pneumonia (VAP) and ventilator-associated tracheobronchitis (VAT). VAP has been described since 1965, while VAT is a relatively newer clinical entity [1,2,3]. US epidemiological studies report an incidence of VAP of 2–16 episodes per 1000 ventilator days [4]. VAT is also a frequent infectious complication of mechanical ventilation with an incidence similar to VAP. Our knowledge regarding the incidence, causative organisms, and impact on patient outcomes of VAT became profound after the early 2000s [2,5,6].

Both VAP and VAT significantly affect patient outcomes because they increase the duration of mechanical ventilation, length of intensive care unit (ICU) stay, similarly to pneumonia, and healthcare costs, provoking antimicrobial resistance due to increased antibiotic consumption [4,5]; VAP has been significantly associated with increased morbidity and mortality in critically ill ICU patients in various studies [4,6,7,8,9,10].

“Ventilator bundles”, targeting the reduction of respiratory infection incidence in the ICU, may significantly decrease the reported rates of infections [11,12]. There are ongoing uncertainties for both infectious entities, especially whether VAP and VAT represent a continuum and, in this respect, whether early management of VAT could prevent VAP as well. Several prevention strategies have been proposed for VAP, and few have been proposed for VAT. These bundles may include avoiding endotracheal intubation itself, restricting sedation use, oral care, enteral nutrition, subglottic secretion aspiration and avoidance of reintubation, and low endotracheal tube cuff pressure [11,12,13,14]. Inhaled antibiotics have also been suggested as a strategy either to adequately manage VAT and prevent its progression to VAP or as a preventive strategy for VAT [15].

Aerosolized/nebulized or tracheobronchially instilled antibiotics have a long history, and they have been evaluated in studies on hospital-acquired respiratory tract infections in the intensive care units (ICUs) since the 1990s [16,17]. Notably, previous studies reported lower incidence to VAP progression when VAT was treated with appropriate antibiotics, emphasising the importance underlining the treatment of both infections [18,19,20]. However, there are still specific questions concerning the efficacy and subsequent emergence of multidrug resistant bacteria.

The purpose of the present narrative review is to describe the role of nebulized antibiotics in the management of ventilator-associated infections in critically ill patients. More specifically, we aimed to review the literature regarding two related questions: (a) whether prophylactic antibiotics administered through the respiratory tract can prevent ventilator associated infections and (b) whether nebulized antibiotic treatment of VAT could affect the potential evolution of VAT into VAP.

## 2. Search Strategy

We used the PubMed, Cochrane Database of Systematic Reviews, and Google Scholar research databases to search for published articles using the following terms: antibiotics AND aerosolized OR nebulized AND ventilator infections. The search was narrowed down to titles and abstracts. Abstracts were then examined, and studies that included the key terms in the title or abstract were considered. The abstracts and/or full texts of selected, potentially relevant papers were further evaluated. The selection was limited to surveys published in English up to 2022. Exclusion criteria for articles included: no accessible full text, publications with only abstracts, and editorials and studies that did not report specific quantitative outcomes.

## 3. Ventilator-Associated Infections: VAP and VAT

Ventilator-associated pneumonia (VAP) may occur in patients who are managed with mechanical ventilation for more than 48 h, and the diagnosis requires radiologic evidence of pneumonia (new infliltrates) and may include the presence of purulent tracheal secretions, fever, signs of inflammatory reaction, respiratory distress, and the presence of microorganisms [9,10]. VAT shares the same diagnostic criteria as VAP, except for the presence of new pulmonary infiltrates; certainly, this cannot rule out misdiagnosed VAP cases if a computed tomography scan could be performed [2,19]. Moreover, oxygenation worsening is usually absent, making VAT a distinct entity on a continuum between lower respiratory tract colonization with potentially pathogenic microorganisms and VAP [21]. Patients developing VAT or VAP may present significant increases in the ventilator, ICU, and hospital days. Compared to VAT, VAP presents more serious morbidity and has been associated with mortality [7,8].

## 4. Rationale for the Use of Aerosolized Antibiotics in the Management of Ventilator Associated Infections

Early and appropriate therapy of VAP has been consistently demonstrated to decrease mortality, but the efficacy of treatment has been challenged by the presence of multidrug-resistant pathogens. More emphasis has been given to the prevention of VAP, instead of VAT, since any preventive measure has been considered cost-beneficial [4]. However, airway colonization, VAT, and VAP are three conditions believed to represent a continuum, with the development of VAP being the most serious complication [22]. It has been suggested that one-fifth of VAT cases progress to VAP during a three day timeframe. This gives the time for etiologic diagnosis of VAT and the opportunity for appropriate treatment administration, which may be translated to ultimate VAP prevention, improved patient outcomes, and reduced healthcare costs. In this respect, a critical point for VAP prevention may be the appropriate management of VAT to avoid progression to VAP, increased MV and ICU days, difficult weaning, or increased costs [23].

Various pathophysiologic mechanisms have been described as potential mechanisms for ventilator-associated infections [24]. Mechanically ventilated patients require orotracheal or nasotracheal or tracheal intubation, which all constitute risk factors for infection per se. As an implanted foreign body, the endotracheal tube (ET) bypasses host defenses and allows inhaled micro-organisms direct access to the airways. Yet, microbial biofilm on the ET surface provides a reservoir of infecting micro-organisms. Along with the biofilm formation within the ET, microaspiration, suppression of the cough reflex, and disturbances in mucociliary clearance are main factors. Additionally, the positive pressure from the ventilator boosts bacteria from the endotracheal tube further into the respiratory tract. Colonisation of the upper respiratory airway may form the basis for respiratory-associated infection of VAT and/or VAP with nosocomial micro-organisms.

Ventilator bundles cannot lead to “zero VAP” due to the fact that microaspiration and biofilm formation cannot be fully prevented, and this is also because systemic antibiotics cannot reach the endotracheal tube and biofilms to kill bacteria. Local application of antibiotics administered through the endotracheal tube might have the advantage of directly presenting the antibiotic in contact with the tubing system, enabling possible penetration into the biofilm. Moreover, the antibiotic is directly administered to the target organ quickly, while intratracheal antibiotic concentrations can greatly exceed the minimum inhibitory concentration of the pathogens. This might enable the eradication of multiple pathogens, including those with difficult-to-treat resistance [25,26,27].

## 5. Methods for Delivery of Antibiotics in the Tracheal Tree

The earliest modality of antibiotic delivery to the tracheal tube, the trachea, and the lung parenchyma was intratracheal installation to the tube (Table 1). Direct intratracheal administration of antibiotics, targeting, among others, the biofilm within the endotracheal tube, increased drug concentrations in bronchial secretions above those in plasma, increasing the bactericidal activity of the antibiotic in the lung [25,26]. The delivery of antibiotics to the tracheal tube and the lungs has been achieved using the available types of aerosol generators, which convert antibiotic solution into small droplets. Several factors and characteristics, involving the nebulizer, ventilator, and medication, must be kept in mind to ensure the effective delivery of nebulized antibiotics to mechanically ventilated patients. Different types of aerosol generators are currently available in the ICUs: jet, ultrasonic, and vibrating mesh nebulizers. The type of nebulizer used may significantly influence the degree of antibiotic penetration in the tracheobronchial tree and the accomplished concentration. Relatively big droplets, with a diameter greater than 5 µm, are mainly deposited in the ventilation circuit and/or the upper airways, median droplets of 3–5 µm are deposited in the proximal bronchi, while droplets of 1–3 µm are deposited in the terminal bronchioles, probably reaching the alveoli [28]. In addition, it should be underlined that the flow speed decreases from the trachea to the terminal bronchioles as the total cross-sectional area increases with each bronchial bifurcation [29].

Jet nebulizers use the already available compressed gas (air or oxygen) from a wall system or the ventilator to produce aerosol. Apart from their low cost, another advantage of jet nebulizers is the capability of synchronization with each breath. Disadvantages include long treatment time, high residual drug volume (0.8–2 mL), possible risk for denaturing medication due to shear stress generated from a highly turbulent flow, and the effect on the tidal volume that the patient receives by the additional delivery of 6–8 L of gas into the ventilator circuit. Generally, it is considered the lowest-efficiency nebulizer [30,31,32]. Ultrasonic nebulizers produce aerosol, creating high-frequency vibrations through piezoelectric crystals. They have the advantage of generating droplets of different sizes, depending on the frequency of vibration. The smaller the particle size—with higher frequency—the deeper they can penetrate the bronchial tree. These nebulizers are considered more efficient than the jet nebulizer, with better treatment time and lower residual volume (0.4–1.2 mL). However, possible overheat of the drug solution after the nebulizer has been in operation for 10–15 min increases the risk of denaturing and possible inactivation of the drug. Another disadvantage is their higher cost [30,31,32].

Vibrating mesh nebulizers generate aerosol by vibration of a plate, producing particles 1–5 μm in size. This nebulizer has the highest efficiency for drug delivery among all others, has a short treatment time, has the lowest residual volume (0.2 mL), and minimally affects the solution temperature and, therefore, the drug inactivation. The only disadvantage is its high cost [30,31,32].

Lung pathophysiology, breathing pattern, and the severity of disease can affect the particle deposition in the lungs. Larger antibiotic particles deposit centrally in the upper respiratory tract, while smaller particles (i.e., <1 μm in size) are more likely to be exhaled [33]. Spontaneous mechanical ventilation modes are associated with turbulent inspiratory flow, reducing drug delivery to the lung. For optimal drug delivery, the use of volume assist-controlled modes, delivering constantly higher tidal volumes and longer inspiratory phase, is preferred, avoiding asynchronies. Adding humidity to the respiratory system promotes mucociliary clearance, preventing airway mucosa dryness and reducing the possibility of bronchospasm. Moreover, if the target of drug delivery is lung parenchyma (for VAP treatment and not VAT/VAP prevention), using heliox—a less dense gas—reduces airflow turbulence, improving antibiotic aerosol penetration in the lungs [34].

The antibiotic selection depends on inherent properties to ensure maximal efficacy and safety. The ideal aerosolized antibiotic should be sterile, nonpyrogenic, preservative-free, with a pH level of 4.0–8.0, and adjusted tonicity and osmolarity of 150–1200 mOsm/L [35]. Therefore, the use of IV drug formulations should be avoided for aerosolization, as the preservatives included might be harmful and associated with increased incidence of bronchospasm.

## 6. Aerosolized or Instilled Antibiotics for the Prevention of Ventilator Associated Infections

Based on data from recent studies, nebulized antibiotics cannot treat VAP or decrease mortality, as shown by the recent Phase 2 and 3 trials (IASIS, INHALE) [36,37]. However, prophylactic antibiotic administration on the tracheobronchial tree could be used to prevent VAP in ICU patients. Intratracheal instillation of antibiotics seems ideal, targeting the prevention of tube biofilm formation or even penetrating the biofilm reducing existing microbes [38]. On the other hand, aerosolized antibiotics—which might improve antibiotic delivery up to the lungs—are mainly targeted to settle on the tracheal epithelium and to kill existing microbes or prevent their growth. Therefore, the scope of antibiotics is to reduce bacterial colonization of the airways as an effective means to prevent VAP. Experimental and clinical studies have dealt with the possibility of prevention of VAP using antibiotics through the trachea. Data are available for specific antibiotics, and this is most likely due to their availability for nebulization and their efficacy in treating respiratory infections due to MDR bacteria.

In a non-human model, the aerosolized kanamycin administered before intratracheal instillation of *K. pneumoniae* prevented the onset of bronchopneumonia, whereas intramuscular administration was not protective. Further experimental studies demonstrated that the endotracheal administration of polymyxin B or gentamicin prevented ventilator-associated pneumonia (VAP), providing the rational for the conduction of clinical studies [39,40].

Polymixin and amikacin often remain the only antimicrobial agents available against *P. aeruginosa* and *A. baumannii*, the most common pathogens causing VAP in Europe and Asia [41,42]. The nebulized administration of these drugs has been advocated to achieve high local concentrations, way above the pathogen MIC. This might be useful in the management of colonization and prevention of VAP [43]. Initial clinical studies evaluated the use of colistin-polymyxin instilled into the trachea or sprayed into the pharynx to prevent Gram-negative bacterial GNB-pneumonia (Table 2). Ruby et al. [44], in a prospective non-randomized study, evaluated the efficacy of intratracheal colistin instillation to prevent nosocomial bronchopneumonia (BPN) in critically ill patients. BPN was assessed in survivors using clinical criteria and microbiological data, while, in non-survivors, it was assessed using histological data. The administration of intratracheal colistin during a two-week period significantly reduced the incidence of Gram-negative BPN; however, digestive selective decontamination was administered additionally to tracheal instillation. Mortality was not significantly influenced. Infections due to colistin-resistant micro-organisms were not observed. A pioneering prospective study [18] evaluated the role of polymixin to prevent nosocomial pneumonia; 744 patients admitted in a respiratory—surgical ICU were randomized to receive polymyxin (2.5 mg/kg BW/day in six divided doses) or a placebo aerosol sprayed into the posterior pharynx and tracheal tube in a mixed population of intubated and non-intubated patients. The incidence of upper airway colonization with *P. aeruginosa* was 1.6% during the polymyxin treatment (total 374 patients) and 9.7% during the placebo cycles (370 patients) (*p* < 0.01). Similarly, three patients in the polymyxin and 17 in the placebo groups acquired *P. aeroginosa* pneumonia (*p* < 0.01). Feeley and co-workers [17] evaluated the efficacy of aerosolized polymyxin B (2.5 mg per kilogram per day) in the prevention of *P. aeruginosa* pneumonia during a seven-month period. The study included 292 intubated and spontaneously breathing patients in a respiratory–surgical ICU. They concluded that daily use of polymyxin B aerosol appears to be a dangerous form of therapy. The mortality rate for acquired pneumonia was 64%, higher than that in previous studies in which no polymyxin was used, although there was only one episode of acquired pneumonia due to *P. aeruginosa*. Moreover, 10 patients acquired pneumonia caused by a polymyxin-resistant organism, while seven cases were caused by organisms not frequently pathogenic to man (*Flavobacteria*, *Serratia*, and *Streptococcus faecalis*). Although these results were not repeated, they prompted the medical community towards skepticism concerning the intratracheal instillation of drugs to prevent VAP.

Klastersky et al. [16] studied the effect of endotracheal gentamicin versus endotracheal placebo in a randomized double-blind study. An amount of 85 comatose patients with tracheostomy were included in the study, and the incidence of tracheal secretion colonization by Gram-negative bacteria was significantly reduced in the gentamicin treatment arm (*p* < 0.01). Moreover, significantly fewer patients suffered bacteriologically confirmed respiratory tract infections (*p* < 0.01). Bacteria isolated from patients treated with gentamicin were slightly more resistant to gentamicin than the microorganisms recovered from control patients, concluding that endotracheal administration of gentamicin may be a helpful adjunct treatment, but special attention should be paid to the possibility for resistance emergence.

In a two-arm, randomized, open-label, controlled trial [6], prophylactic nebulized colistin was used in a total of 168 patients in an ICU where VAP, due to MDR Gram-negative bacilli, was prevalent. The colistin (Col) group received prophylaxis with 500,000 U colistimethate sodium (45.5 mg colistin base activity), and the NS group received placebo saline. The median time of treatment initiation was, 6.5 (4–9.75) and 7 (4–10) h in the control group, following intubation, and the treatment was administered three times daily for the first 10 ICU days or until extubation. The first positive TBA culture was detected on 10 (5–17) vs. 4 (2.5–8) ICU day in the Col and NS groups, respectively. Although fewer subjects developed VAP in the intervention group, the difference was not statistically significant (16.7% versus 29.8%, respectively, *p* = 0.07), at the 30-day follow up. Regarding the secondary outcomes, the intervention resulted in a lower VAP incidence density rate (total number of respiratory infection cases per 1000 ventilation days) (*p* < 0.01) and less VAP episodes either from Gram-negative bacteria (*p* = 0.03) or MDR bacteria (*p* = 0.04). There was also no development regarding ICU or hospital mortality. However, among all patients who developed VAP (n = 39), those having received prophylactic nebulized colistin presented a significantly lower ICU mortality (*p* = 0.016). The authors hypothesize that the control of airway inflammation during the VAP episode may explain the survival benefit. This is also supported by the findings of lower SOFA score on the infection day and the white blood cell count following VAP diagnosis in the Col group vs. the NS group. In this study, neurosurgical admission was a risk factor for GNB-VAP, whereas GNB-VAP was lower in the neurosurgical Col group. Thus, the prophylactic administration of inhaled colistin may become attractive, especially in neurocritical patients. Interestingly, during the study period (19 months), there was no difference in the development of colistin-resistant bacteria or multidrug resistance between the two groups.

Povoa and colleagues [45] conducted a meta-analysis on the prophylactic use of nebulized antibiotics and included five of the above comparative randomized trials, representing data from approximately 1000 mechanically ventilated patients receiving prophylactic antibiotics. The antibiotics were administered either by nebulization or intratracheal instillation. The dosage, duration, route of antibiotic administration, the VAP occurrence, VAP due to multidrug-resistant (MDR) pathogens, and the ICU mortality were taken into account. In three studies [6,46,47], the antibiotic was administered in the form of aerosolized preparation, whereas, in the remaining two studies, tracheal instillation was performed [16,44]. The authors concluded that prophylactic antibiotics administered through the respiratory tract reduced the occurrence of VAP when compared to placebo or no treatment (OR 0.53; 95% CI 0.34–0.84), but this effect was seen when the antibiotics were given by nebulization (OR 0.46; 95% CI 0.22–0.97) and not when administered by intratracheal instillation (OR 0.57; 95% CI 0.28–1.15). The weighted pooled proportion (meta-proportion) for VAP occurrence was 32% (*p* = 0.02), and the pooled odds ratio (meta-odds ratio) for developing pneumonia was 0.53 compared to control. The antibiotic prophylaxis did not increase the development of VAP due to MDR pathogens. The ICU mortality was not different between the compared groups with or without prophylaxis. It is evident that, if this treatment modality is selected, the antibiotics should be delivered by aerosolization to achieve more uniform distribution and deeper penetration into the lung parenchyma [26]. Whether ultrasonic or vibrating mesh nebulizers are superior to jet nebulizers remains to be proved. In this respect, the use of aerosolized antibiotics for the prevention of VAP appears an attractive option, but the evidence for its efficacy is still inconclusive.

Others studied aerosolized cephalosporines administered through the respiratory tract–endotracheal tube. Wood et al. [46] evaluated the safety and efficacy of aerosolized ceftazidime for the prevention of VAP in forty critically ill trauma patients. Moreover, they evaluated the drug effects on the proinflammatory response. Within 48 h of ICU admission, the patients were randomly assigned to receive aerosolized ceftazidime 250 mg every 12 h or placebo (normal saline) for up to seven days. VAP frequency was decreased without adversely affecting the ICU flora. In detail, the frequency of VAP in patients receiving aerosolized ceftazidime was 73% lower than that in patients receiving placebo at ICU day 14 (15% vs. 55%, *p* = 0.021), and it was 54% lower for the entire ICU stay (30% vs. 65%, *p* = 0.022). No clinically significant changes in bacterial culture and sensitivity patterns were observed. In addition, the proinflammatory response in the lung was attenuated; the frequency of VAP was directly related to changes in TNF-alpha and IL-beta. In addition, the subjects receiving empiric antibiotics received less systemic antibiotics compared with those receiving placebo. In another single-institution double-blind, randomized trial [47], the patients were randomized to receive a seven-day course of aerosolized ceftazidime or placebo (52 patients in the placebo arm and 53 patients in the ceftazidime arm), and VAP rate was not reduced, but neither was the incidence of other infectious complications increased, or the development of MDR bacteria in either group, at two weeks and 30 days. The patients had received either nebulized ceftazidime or placebo every 12 h for 7 d or until the subject was either extubated or removed from the ventilator, while factors, such as the mechanism of injury, presence of spinal cord injury, and the number of blood products received, were considered. Adair et al. [48] compared the efficacy of nebulized gentamicin to the parenteral cefotaxime or parenteral cefuroxime in preventing the formation of ET biofilm. Nebulized gentamicin attained high concentrations in the ET lumen and was more effective in preventing biofilm formation than parenterally administered cephalosporin, concluding that this method may be effective in preventing VAP.

More recently, inhaled specific polyclonal antibodies have been used to protect against tracheal colonization by specific microbes, and specifically *P. aeruginosa* in experimentally model, which may be an alternative safe protection method for VAP in the future [49]. The possibility to administer monoclonal antibodies against specific pathogens, is an exciting alternative to reduce colonization of tracheobronchial tree and prevent–or even treat-VAP and VAT. Polyclonal Pa-IgY antibodies were administered in a murine pneumonia model and prophylactic properties were primarily reported; the pulmonary bacterial load was also reduced with post-exposure treatment [50].Similarly, the prophylactic properties of Pa-IgY in a non-human model of VAP development was investigated. Twelve pigs were mechanically ventilated, and allocated to either receive nebulized *P. aeruginosa* or nebulized *P. aeruginosa* + specific polyclonal anti-*P. aeruginosa* IgY antibodies. Tracheal growth of *P. aeruginosa* increased in both groups during the experiment, but with lower growth in the Pa-IgY-treated group. After 12 h, the treatment effect was diminished and bacterial growth increased in both groups. However, in this porcine model, Pa-IgY antibodies decreasd the airway bacterial colonization, although transiently, leaving a promising potential for the future [50].

**Table 2:** Clinical studies examining the effectiveness of nebulized antibiotics administered pre-emptively to prevent VAP/VAT episodes and/or colonization.

Author (Country, Year)	Design and Setting	Patients	Drugs and Method of Nebulization	Findings	Limitations
Klastersky, Belgium, 1974 [16]	Double blind, placebo controlled	85	Gentamicin 80 mg, tid, intratracheal injection	Purulent secretions (gentamicin vs. placebo): 87.7% vs. 94.8%, *p* < 0.02 Positive tracheal aspirates: 56.5% vs. 79.3%, *p* < 0.01 **Pulmonary infection:** 11.6% vs. 40.4%, *p* < 0.01	Single center Non-aerosolized aministraion of antibiotic
			Placebo 2 mL of N/S, tid, intratracheal injection	Deaths probably resulting from infection: 26% vs. 37.5%	
			Duration: UNK (seven and five patients were treated for more than 30 days)	**Resistance emergence:** isolates slightly more resistant in gentamicin group compared to placebo	
Klick, J.M., USA, 1975 [18]	Prospective, placebo- controlled During alternating peiods of	655	Polymyxin 2.5 mg/k/day (6 doses daily) aerosolized antibiotic in the posterior pharynx and tracheal tube	**Colonization:** Upper airway (polymyxin vs. placebo): *P. aeroginosa*: 1.6% vs. 9.6%, *p* < 0.01 Enterobacteriae: 18.7% vs. 6.8%, *p* < 0.01 Staph aureus: 2.4% vs. 2.8%, *p* = NS	Single center Comparison between periods of different treatments (non-synchronous)
			Placebo N/S		
			Duration: alternating cycles of eight weeks during which the same treatment was administered to all patients	Pneumonia: 9% vs. 5.6%, *p* < 0.01	
Feeley, T.W., USA, 1975 [17]	Prospective, non-comparative	292	Polymyxin 2.5 mg/kg/day (n six doses)	**Colonization:** GNB: An amount of 69 patients (90 episodes) (24%) Polymyxin R: An amount of 67/90 (74%) GPB: An amount of 48 patients (16%) Fungi: An amount of 40 patients (14%)	Single center Absence of control arm Non-aerosolized aministraion of antibiotic
			Non-intubated patients: polymyxin sprayed in the phranx	**Pneumonia:** Eleven patients (10 polymyxin R)–incidence 3.8% An amount of 10/11 patients had prior colonization (same pathogen).	
			Intubated patients: polymyxin injected into the tracheal tube	**ICU LOS:** No microorganism: five days 1 GNB: 7.2 days ≥2 GNB: 11.8 days	
			Duration: ICU stay (mean—five days)	**Mortality:** General: 12% VAP patients: 64%	
Rouby, J.J., France, 1994 [44]	Prospective, non-randomized, comparative	598	Colistin 1,600,000 IU/day (eight doses/24 h)	Bronchopneumonia Survivors: 37% coli(−) vs. 27% coli(+), *p* < 0.01 Non-survivors: 61% coli(−) vs. 36% coli(+), *p* < 0.001 bronchopneumonia from Gram(+): 18% coli(−) vs. 30% coli(+), *p* < 0.01	Single center Non-randomized Non-aerosolized aministraion of antibiotic Use of digestive selective decontamination
			Intratracheally injected		
			Duration: 15 days	**Resistance emergence:** 55% coli(−) vs. 67% coli(+), *p* = NS	
Wood, C.G., USA, 2002 [46]	Prospective, randomized, placebo-controlled	40	Aerosolized ceftazidime 250 mg bid	VAP by D7 (ceftazidime vs. placebo): 10% vs. 26%, non significant difference VAP by D14: 16% vs. 56%, *p* = 0.021 VAP through ICU stay: 32% vs. 68%, *p* = 0.022	Single center Limited number of patients
			Placebo	Antibiotic duration (for documented infections): 6 ± 8 vs. 13 ± 11, *p* = 0.024 MV duration: 16 ± 11 vs. 18 ± 13, *p* = NS ICU LOS: 19 ± 11 vs. 21 ± 12, *p* = NS	
			Duration: seven days	**Resistance emergence:** non-different resistance patterns compared to historical controls	
Adair, C.G., UK, 2002 [48]	Prospective, non-comparative	36	Aerosolized gentamicin 80 mg, tid (12 patients)	Biofilm formation: 41.6% vs. 100% vs. 100%	Single centre, Lack of VAP estimaton
			Parenteral cefotaxime (12 patients)	Tracheal antibiotic concentrations: Gentamycin >>> MIC, cefotaxime < MIC, cefuroxime < MIC	
			Parenteral cefuroxime (12 patients)		
			Duration: covered intubation period (six days) [(2–32) vs. (2–13) vs. (2–15), respectively]		
Claridge, J.A., USA, 2007 [47]	Double-blind, randomized, placebo controled	105	Aerosolized ceftazidime 250 mg, bid	VAP D15: 46% vs. 40%, *p* = 0.5 VAP D30: 50% vs. 49% MDR VAP: 28% vs. 23%, *p* = NS MDR infections: 34% vs. 23%, *p* = NS	Single center Small size
			Placebo		
			Duration: seven days or until extubation		
Karvouniaris, M., Greece, 2015 [6]	Single center, two-arm, randomized, open-label, controlled	168	Aerosolized colistin 500.000 IU, tid	Positive TBA (colistin vs. placebo): 71.4%vs. 83.3% Isolation day: 10 vs. 4, *p* < 0.01	Single center Open label
			Placebo: N/S		
			Duration: 10 days	VAP incidence: 16.7% vs. 29.8%, *p* = 0.07 Incidence density rate: 11.4 vs. 25.6, *p* < 0.01 VAT incidence: 6% vs. 7.1%, *p* = NS Insidence density rate: 4.1 vs. 6.6, *p* < 0.01 ICU mortality: 29.8% vs. 34.5%, *p* = 0.62 ICU mortality (VAP patients): 7.1% vs. 44%, *p* = 0.028 Resistance emergence: no difference between groups	

Bid: two times daily; GB: Gram-negative bacteria; ICU: intensive care unit; LOS: length of stay; MDR: multi-drug resistant; MV: mechanical ventilation; NS: non-significant, N/S: normal saline; TBA: transbronchial aspirate; tid: three times daily; UNK: unknown; VAP: ventilator-associated pneumonia, VAT: ventilator-associated tracheobronchitis.

## 7. Aerosolized Antibiotics as a Monotherapy for Ventilator-Associated Tracheobronchitis

Aerosolized antibiotics (AA) as a monotherapy for VAT have been investigated in few original studies, as shown in Table 3. Palmer et al. [51] found that, in critically ill patients with VAT, AA may have favorable outcomes in the resolution of VAP signs and symptoms, and it may also decrease systemic antibiotic use, reduce bacterial resistance, and facilitate weaning. Maskin et al. found that low-dose inhaled colistin methylsulfate [52] decreased the volume, purulence, and bacterial load in tracheal secretions in the vast majority of patients with VAT due to multidrug-resistant Gram-negative bacteria (MDR-GNB), which may be translated into a reduced the risk of subsequent VAP development. Actually, only one patient subsequently developed VAP. Palmer et al., in a more recent study [53], found that, among 47 patients, AA eradicated the original resistant organism and MDR bacteria on culture compared to placebo. Additionally, fewer resistant organisms were seen compared to the placebo group, and the intervention significantly reduced mCPIS, secretions volume, and ventilator days in the treatment group. A large French multicenter study (AMIKINHAL study, ClinicalTrials.gov. NCT03149640) evauated the efficacy of a three-day course of inhaled amikacin to placebo in preventing VAP, among 850 patients, under invasive mechanical ventilation. The results will be available in 2023.

The paucity of available data has been underlined in the literature. Systematic reviews of published data do not clarify further this issue. Russell et al. [54] suggested that the available evidence to support the use of aerosolized antibiotics, either delivered solely or concomitantly with systemic antibiotics, is not sufficient. This was based on the observation that, in most studies, aerosolized antibiotics are not used as a single treatment regime. Additionally, there were significant differences concerning the methods used for aerosolized antibiotic delivery (e.g., ultrasonic or vibrating plate nebulizers), the study protocols, and outcome definitions. In a review and meta-analysis [55] performed on adults under mechanical ventilation, nebulized antibiotics significantly decreased the emergence of antibiotic resistance in VAT patients (RR, 0.18; 95% CI 0.05–0.64; I 2.0%). However, the mortality and days on ventilator did not decline. The overall rate of respiratory complications observed was 9%. The authors emphasize the need for RCT-aerosolized antibiotic treatment, with an emphasis on homogenized criteria for population inclusion, antibiotic delivery methods, and safety issues. Another review and meta-analysis, analyzing 12 studies (2 regarding VAT), suggested that inhaled colistin monotherapy should be thoroughly evaluated in the treatment of respiratory tract infections due to MDR *P. aeruginosa* and *A. baumannii* [22]. Larger studies are warranted to determine whether patients with VAT should receive systemic and/or aerosolized antimicrobials in order to prevent VAP in critically ill patients [5]. Moreover, Agrafiotis et al. showed that the frequency of VAT was 11.5%, but the subsequent transition to VAP decreased significantly only in patients receiving aerosolized antimicrobial agents [55].

Actually, the guidelines and position paper from the European Society of Clinical Microbiology and Infectious Diseases (ESCMID) on the use of nebulized antimicrobials for the treatment of respiratory infections in adult patients under mechanical ventilation recommended to avoid the additional use of aerosolized antibiotics for the treatment of VAT because the quality of available data is rather low [56].

**Table 3:** Studies examining the effectiveness of nebulized antibiotics as the only therapeutic measure in the prevention of the evolution of VAT to VAP.

Author (Country, Year)	Design and Setting	Patients	Drugs and Method of Nebulization	Findings	Limitations
Palmer L.B., USA, 2008 [51]	Double-blind, randomized, placebo-controlled study	43	Vancomycin 120 mg/8 h or gentamicin 80 mg/8 h in 2 mL normal saline every 8 h for 14 days) or nebulized placebo. Similar systemic antibiotics were given in both groups.	Neulisation vs. placebo group Criteria for VAP diagnosis (CDC-NNIS criteria) s at day 14 in 36% in the nebulization group vs. 79% in the placebo group Resistance emergence 0/19 vs. 8/24, *p* = 0.0056	Single center comparison with contrls from other studies
				WBC day 14: 9.2 ± 3.3 vs. 14.9 ± 8.1, *p* = 0.016 New antibiotic initiation 8/19 vs. 17/24, *p* = 0.042 Weaning: 84.2% vs. 54.1%, *p* = 0.052 Mortality: 4/19 vs. 4/24, *p* = 0.999	
Palmer, L.B. USA, 2014 [53]	Double-blind placebo-controlled study	47	Vancomycin 120 mg every 8 h for 14 days and or Aminoglycoside (gentamicinsulfate,80 mg every 8 h, or amikacin,400 mg every 8 h) dissolved in N/S 0.9% to achieve volume of 2 mL vs. placebo via a jet nebulizer for 7 days. Similar amounts of appropriate systemic antibiotics were given in both groups	AA eradicated the original resistant organism at EOT in 26/27 pts compared with 2 of 23 for placebo AA eradicated 14 out of 16 for MDRO patients compared with 1 of 11 for placebo. More newly resistant organisms were seen in the placebo group. AA significantly reduced mCPIS, secretions volume, and ventilator days in the treatment group.	Non-formal quantitative cultures Eradication of bacteria at EOT could be attributed to in vitro suppression of bacterial growth and not death. Difference in baseline APACHE scores.
Maskin L.P., Argentina, 2015 [52]	Prospective observational study of inhaled colistin in patients with VAT due to MDR-GNB	20	Colistimethate sodium (CMS) 625.000 IU dissolved in 3 N/S 0.9% to achieve volume of 6 mL every 8 h via a vibrating mesh nebulizer for seven days	Decreased tracheal secretion volume, purulence and bacterial load. Negative aspirates culture (eradication) at day 7 for 95% (19/20) of the pts. CPIS score improvement. One patient subsequently developed VAP.	Absence of control arm. Single center study
Athanassa Z.E., Greece, 2014 [57]	Prospective observational study of inhaled CMS in patients with VAT	20	1 MU (80 mg) dissolved in N/S 0.9% to achieve volume of 9 mL every 8 h for seven days.	Cure in 16 of 20 patients (80%), microbiological response in 12 (all GNB), [eradication in eight and bacterial growth decline in four]	Not designed to evaluate VAP prevention

AA: aerosolized antibiotics; APACHE: Acute Physiology And Chronic Health Evaluation; CDC NNIS: Centers for Disease Control National Nosocomial Infections Surveillance; CPIS: Clinical Pulmonary Infection Score: EoT = end of treatment, MDR-GNB = multi drug resistant Gram-negative bacteria, MDRO: multi-drug resistant organisms; MV: mechanical ventilation; VAP: ventilator-Aassociated pneumonia; VAT = ventilator-associated pneumonia.

## 8. Adverse Effects of the Aerosolized Antibiotic Therapies

The emergence of MDR bacteria, following tracheobronchial instillation/aerosolization of antibiotics, is a major concern when implementing strategies using antibiotics to prevent infections. Resistance emergence has not been commonly identified in the studies using aerosolized antibiotics. Karvouniaris et al. reported a reduced risk for VAP due to MDR pathogens when nebulized colistin was used (OR 0.33 CI 95% 0.12–0.88) [6]. In accordance with the above investigation, in a double-blind placebo-controlled study [51], critically ill intubated patients were randomized to receive aerosolized antibiotics or placebo for the treatment of respiratory infections aiming to decrease the need for systemic antibiotic. Of the subjects receiving the aerosolized antibiotics, 13% developed new antibiotic resistance compared to 55% in the placebo group. New drug resistance to aerosolized antibiotics was not seen. In this respect, the presented data do not support the hypothesis that this type of antibiotic therapy presses the emergence of resistant bacteria.

However, concerns regarding the emergence/worsening of antibiotic-resistance when antibiotics are widely used in ICU critically ill patients still remain. The concerns are further implicated when considering the strict administration criteria of inhaled antibiotic strategies, such as optimal formulations, as well as length and dosage of treatment regimens. Moreover, whether ultrasonic, vibrating-mesh, or jet nebulizers in mechanically ventilated patients are preferred has not yet been determined. Furthermore, the pharmacokinetics/pharmacodynamics of inhaled therapies might be challenging. International scientific societies remain skeptical for the use of inhaled antibiotic strategies. The American Thoracic Society guidelines for the management of adults with hospital-acquired and ventilator-associated pneumonia do not recommend the use of aerosolized antibiotics to prevent VAP [58], and the Centers for Disease Control and Prevention discourage this strategy as well [59]. Moreover, the ESCMID recommended avoiding the use of aerosolized antibiotics either to prevent or treat VAP in mechanically ventilated adults due to the lack of strong evidence for their efficacy and the risk of toxicity [56].

The presence of adverse effects associated with the use of aerosolized antibiotics is another concern. Irritation and airway inflammatory reaction may lead to cough, bronchospasm, bronchoconstriction-wheezing, desaturation, and even hypoxemia. Among inhaled antibiotics, colistin is more responsible for these effects [60,61]. The preceding administration of an aerosolized β-agonist might decrease the incidence of bronchospasm occurrence. However, if this strategy is unsuccessful, aerosolized antibiotics may have to be discontinued. Occlusion of the ventilation circuit, usually resulting from obstruction of the expiratory filter, may have very serious consequences, including cardiac arrest. Therefore, monitoring of the peak airway pressure during antibiotic administration is valuable, and the expiratory filter could be exchanged after each treatment session [60,62].

## 9. Conclusions

Regarding our view, the role of antibiotic aerosolized therapy to manage ventilator associated infections, especially those due to MDR Gram-negative bacteria is promising, but clinical data are limited, and there are still questions that require further research in order to be answered. This is most important when considering the marked heterogeneity in clinical practice, with significant differences in the use of aerosolized antibiotics between patients. First, large randomized controlled trials should be conducted to confirm the benefits of AA and to explore the impact on antibiotic selection pressure with the use of aerosolized antibiotics. Research may also need to focus on early (pre-emptive) treatment of VAT to prevent evolution to VAP, using appropriate nebulized antibiotics, especially in those at high risk for VAP development. Moreover, future research should focus how to refine aerosolized treatment in terms of delivery device technology, adequate antibiotic dosing, and specific protocol design.

## Figures and Tables

**Table 1:** Methods of antibiotic delivery in the tracheobroncial tree.

	Aerosol Generation	Advantages	Disadvantages
Direct tracheal instillation	None	Easy to perform	Restricted time of antibiotic contact to the tracheal tube and upper tracheobronchial tree
		No cost	
Jet	Compressed gas (air or oxygen)	Low cost	Long treatment time High residual drug volume (0.8–2 mL) Risk of denaturing the medication (fighly turbulent flow) Increase in delivered tidal volume
		Synchronisation with each breath	
Ultrasonic	High frequency vibrations through piezoelectric crystals	Generate droplets of different sizes (higher frequency-smaller droplets-deeper penetration in the lung parenchyma)	Intermeddiate cost Overheating of the solution (after the nebuliser is in use for 10–15 min) Posible denaturing/drug inactivation.
		Treatment time	
		Lower residual volume (0.4–1.2 mL)	
Vibrating mesh	Vibration of a plate Partice generation of 1–5 μm in size	Short treatment time, lowest residual volume (0.2 mL)	High cost
		Minimal change in solution temperature (less likely to denature and inactivate the drug).	

The methods are presented in terms of efficacy. The first means of antibiotic administration is the least effective, and the last is the most effective.

## Data Availability

Not applicable.
